# Association Between Copeptin and Six-Month Neurologic Outcomes in Patients With Moderate Traumatic Brain Injury

**DOI:** 10.3389/fneur.2021.749110

**Published:** 2022-04-25

**Authors:** Jin Pyeong Jeon, Seonghyeon Kim, Tae Yeon Kim, Sung Woo Han, Seung Hyuk Lim, Dong Hyuk Youn, Bong Jun Kim, Eun Pyo Hong, Chan Hum Park, Jong-Tae Kim, Jun Hyong Ahn, Jong Kook Rhim, Jeong Jin Park, Heung Cheol Kim, Suk Hyung Kang

**Affiliations:** ^1^Department of Neurosurgery, Hallym University College of Medicine, Chuncheon, South Korea; ^2^Department of Orthopaedic Surgery, Hallym University College of Medicine, Chuncheon, South Korea; ^3^Institute of New Frontier Research, Hallym University College of Medicine, Chuncheon, South Korea; ^4^Department of Neurosurgery, Jeju National University College of Medicine, Jeju, South Korea; ^5^Department of Neurology, Konkuk Medical Center, Seoul, South Korea; ^6^Department of Radioilogy, Hallym University College of Medicine, Chuncheon, South Korea

**Keywords:** biomarkers, traumatic brain injury, copeptin, outcome, cohort study

## Abstract

**Background:**

Copeptin has been reported as a predictive biomarker for the prognosis after traumatic brain injury (TBI). However, most of them were in patients with severe TBI and limited value in predicting outcomes in patients with moderate TBI defined as Glasgow Coma Scale (GCS) score from 9 to 12. We aimed to investigate the predictive value of copeptin in assessing the neurologic outcome following moderate TBI.

**Methods:**

Patients were prospectively enrolled between May 2017 and November 2020. We consecutively measured plasma copeptin within 24 h after trauma, days 3, 5, and 7 using ELISA. The primary outcome was to correlate plasma copeptin levels with poor neurologic outcome at 6 months after moderate TBI. The secondary outcome was to compare the prognostic accuracy of copeptin and C-reactive protein (CRP) in assessing the outcome of patient.

**Results:**

A total of 70 patients were included for the final analysis. The results showed that 29 patients (41.4%) experienced a poor neurologic outcome at 6 months. Multivariable logistic regression analysis revealed that increased copeptin (odds ration [OR] = 1.020, 95% *CI*: 1.005–1.036), GCS score of 9 or 10 (*OR* = 4.507, 95% *CI*: 1.266–16.047), and significant abnormal findings on CT (*OR* = 4.770; 95% *CI*: 1.133–20.076) were independent risk factors for poor outcomes. Consecutive plasma copeptin levels were significantly different according to outcomes (*p* < 0.001). Copeptin on day 7 exhibited better prognostic performance than CRP with an area under receiver operating characteristic curve (AUROC) difference of 0.179 (95% *CI*: 0.032–0.325) in predicting 6-month poor outcomes.

**Conclusion:**

Plasma copeptin level can be a useful marker in predicting 6-month outcomes in patients with moderate TBI.

## Introduction

Traumatic brain injury (TBI) is a dynamic disease that can be rapidly exacerbated. In addition to initial direct trauma to the brain, secondary brain injury due to inflammation, disruption of blood-brain barrier (BBB), oxidative stress, and metabolic disturbance worsens the condition of the brain (1, 2). Accordingly, early detection of disease progression and exacerbation in high-risk TBI patients is critical for a good neurologic outcome. Many studies have been conducted to find biomarkers that predict disease progression following TBI. However, TBI is a fairly complex disease and the prognosis is largely determined by the initial injury severity. Thus, it may be difficult to accurately determine the usefulness of biomarkers due to heterogeneity and TBI severity. To study biomarkers that predict the disease prognosis following TBI, it is necessary to adjust for factors that greatly influence the outcome and the results should be analyzed differently according to the TBI severity. Severity of trauma is categorized into three groups by the score of Glasgow Coma Scale (GCS), mild (13–15), moderate (9–12), and severe TBI (3–8).

Few studies have been conducted with moderate TBI as the main focus. We believe that this is because the number of patients with moderate TBI is smaller than that of mild TBI in actual clinical filed and the clinical significance of moderate TBI is perceived to be less important than that of severe TBI. Patients with moderate TBI have more abnormal radiological findings on CT than those with mild TBI. However, they do not require surgical interventions as in those with severe TBI. In addition, most patients with moderate TBI have a tendency to expect good outcomes clinically. However, in the case of moderate TBI in the actual medical field, many cases show worsening of TBI. Thus, attention should be paid to it as much as severe TBI due to its clinical significance. Einarsen et al. (3) reported that more than 80% of the patients with moderate TBI had abnormal findings on CT and 60% of them were treated in the intensive care unit (ICU). Compagnone et al. (4) reported that the rate of emergency surgery was 22% and the rate of delayed surgery was 14% following moderate TBI. Therefore, it is necessary to monitor moderate TBI strictly using a robust biomarker for early detection of patients with a high risk of exacerbation (4).

Among various biomarkers, copeptin which is the C-terminal part of arginine vasopressin (AVP) is considered to be a product of the stress response of hypothalamus-adrenal axis after TBI and thought to be a specific biomarker related to TBI severity and prognosis (5–7). A recent meta-analysis (8) revealed that increased plasma copeptin levels was independent risk factor for poor neurologic outcomes [standard mean difference (SMD) = 1.44; 95% *CI*: 1.20–1.68] and mortality (SMD = 1.37; 95% *CI*: 1.16–1.58). However, most of the studies used in the meta-analysis targeted patients with severe TBI, thus, there can be a limit to apply the results in patients with moderate TBI. Here, we consecutively measured plasma copeptin and compared its association with the outcome to demonstrate whether copeptin can be reliable prognostic factor in predicting outcomes in patients with moderate TBI.

## Materials and Methods

### Study Population

This study was approved by the Institutional Review Board (No. 2016-3 and No. 2019-06-006) as a retrospective cohort study. Written informed consent was received from the patients or their relatives. This study was based on data from five university hospitals that have prospectively collected data of brain diseases, such as TBI and stroke by the establishment of cohort since 2015 (9). Among these patients, we enrolled patients with TBI to assess the association between copeptin and outcomes for patients with moderate TBI from May 2017 to November 2020. The inclusion criteria were as follows: (1) patients with moderate TBI with a GCS score of 9–12 at initial neurologic examination; (2) adult patients 18 years of age or older; and (3) patients who were hospitalized for TBI. We excluded patients with following conditions: (1) severe TBI patients with a GCS score ≤ 8; (2) patients who did not receive hospitalization; (3) in case of accompanying spinal cord injury, penetrating injuries, or other injuries requiring surgical intervention; (4) when patient follow-up was not possible; (5) insufficient medical records; (6) GCS score were not accurately checked with drugs used in other hospitals; and (7) patients with severe systemic inflammation within 72 h due to exacerbation of existing medical conditions (4).

### Clinical Assessments

For statistical analysis, the GCS score in patients with moderate TBI was divided into two groups of low GCS score (9 or 10) and high GCS score (11–12) (4). Neuroworsening was defined as the GCS score decreased by more than 2 points compared with that at the time of admission, motor GCS score decreased by more than 1 point, or changes in pupil size or pupil light reflex after admission concomitant with worsening findings on CT (e.g., increased hematoma and progression of cerebral edema) (4, 10). Based on CT findings, it was classified whether there was a significant abnormal findings or not. Significant abnormal findings were defined as diffuse injury type III and IV with intracranial hematoma (4). In addition, laboratory results and accompanying physical injuries were reviewed.

### Study Outcomes

The primary outcome was to correlate plasma copeptin levels with poor neurologic outcome at 6 months after TBI. Poor outcome was defined as Glasgow Outcome Scale Extended (GOSE) score ≤ 6, indicating moderate, severe disability, vegetative state, or death (3). The secondary outcome was to compare the prognostic accuracy of copeptin and C-reactive protein (CRP) in predicting the outcome of patient since CRP is known to be associated with TBI outcome and can be easily measured in hospitals (11, 12).

### Copeptin Measurement

Venous blood samples were collected within 24 h after trauma on 3rd, 5th, and 7th day. The blood samples were directly transferred into serum separator tubes and allowed to clot overnight at 4°C, before centrifugation at 1,000 x g for 15 min. The plasma samples were removed and stored at −70°C in a deep freezer until assayed. The optical density was measured at 450 nm using a GloMAX Discover System (Promega, WI, USA). The plasma copeptin levels were determined using an ELISA assay (CUSABIO, Wuhan, China) according to the instructions of manufacturer (12).

### Statistical Analysis

Chi-square or Fisher's exact test was performed for categorical variables and expressed as number of subjects and percentage. Paired *T*-test or Student's *t*-test was used for continuous variables and expressed as mean ± SD. Copeptin and CRP levels are presented as median and interquartile range (IQR). Mann–Whitney *U*-test was used for comparing copeptin and CRP levels based on the neurological outcome (13). Cohen's kappa coefficient was used to calculate the degree of agreement between investigators (Supplementary Table S1) (14). Univariate analysis was performed to find relevant factors for neurologic outcomes following moderate TBI. A multivariable logistic regression analysis was performed further to confirm the statistical independence of the variables with *p* < 0.10 (15). Repeated measures ANOVA and Mauchly's test of sphericity were carried out to assess the relationship between consecutively measured copeptin and CRP and neurologic outcomes (16). A receiver operator characteristic (ROC) curve was made to determine the optimal cut-off value for predicting the poor outcome. The comparison of outcome prediction between copeptin and CRP was performed using the area under ROC curve (AUROC) (12, 17). The *p* < 0.05 was regarded as statistically significant. Statistical analyses were done by SPSS V.25 (SPSS, IL, USA) and MedCalc (www.Medcalc.org).

## Results

### Clinical Characteristics of Patients

**Figure 1:**
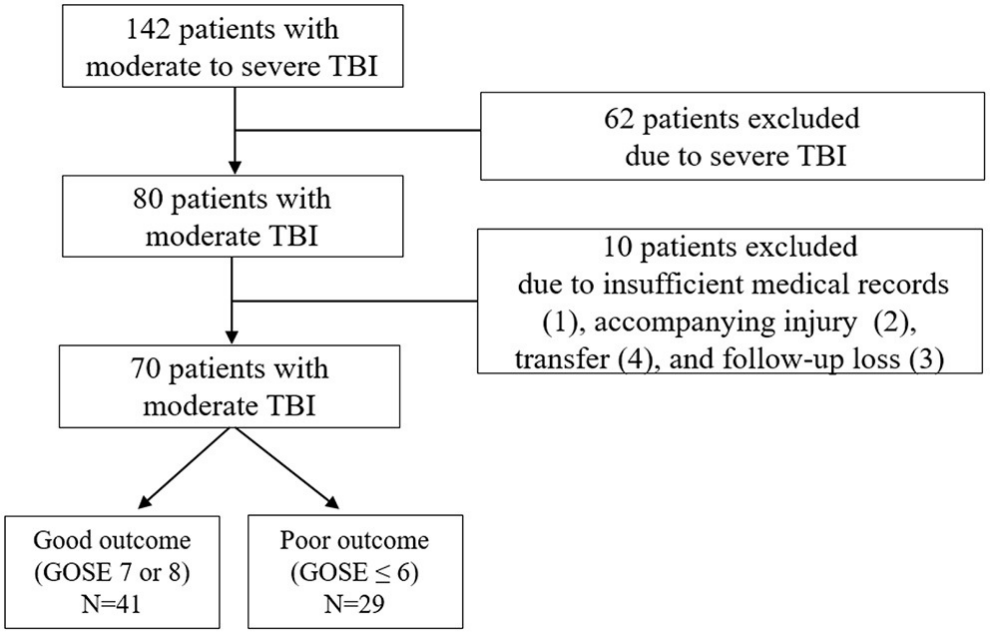
Flow diagram of the study. GOSE, Glasgow Outcome Scale Extended; and TBI, traumatic brain injury.

A total of 142 patients with moderate to severe TBI were initially enrolled. After exclusion of severe TBI (*n* = 62) and moderate TBI with insufficient medical record (*n* = 1), accompanying injury (*n* = 2), transfer (*n* = 4), and follow-up loss (*n* = 3), remaining 70 patients with moderate TBI were finally included for the analysis (Figure 1). After 6 months, 29 patients (41.4%) were observed to have a poor neurologic outcome defined as GOSE <6. The Cohen's kappa for the interpretation of the CT findings including abnormal findings and diagnosis was 0.857, suggesting almost perfect agreement (Supplementary Table S1). Mean age of the enrolled patients was 61.5 ± 18.9 years and 56 patients (80.0%) were men. Intracranial findings on CT were as follows: subdural hemorrhage (*n* = 19, 27.1%), epidural hemorrhage (*n* = 10, 14.3%), traumatic subarachnoid hemorrhage (*n* = 11, 15.7%), and contusion (*n* = 5, 7.1%). Overall, 64.3% of the patients with moderate TBI had intracranial lesions on CT. When comparing patients with good outcome and with poor outcome, there was no significant differences in gender, age, and underlying diseases, such as hypertension, diabetes mellitus, hyperlipidemia, and smoking (Table 1). Univariate analysis showed that poor outcome was associated with GCS score of 9 or 10 (*p* < 0.001), significant abnormal findings on initial CT (*p* < 0.001), neuroworsening (*p* = 0.005), and craniotomy and hematoma removal (*p* = 0.005). Moderate TBI patients with poor outcome [327.2 (290.2–349.9) pg/ml] had significantly higher level of plasma copeptin than those with good outcome [285.9 (254.6–313.2) pg/ml; *p* < 0.001]. The CRP levels were increased in patients with poor outcome [17.3 (11.8–21.3) mg/L] than those with good outcome [14.0 (11.0–16.4) mg/l; *p* = 0.019]. Multivariable logistic regression analysis demonstrated that increased copeptin (*OR* = 1.020, 95% *CI*: 1.005–1.036), GCS score of 9 or 10 (*OR* = 4.507, 95% *CI*: 1.266–16.047), and significant abnormal findings on initial CT (*OR* = 4.770; 95% *CI*: 1.133–20.076) were significantly associated with poor outcomes in patients with moderate TBI (Table 2).

**Table 1:** Differences in clinical characteristics, neurologic and radiological findings, laboratory results, and treatment in patients with moderate traumatic brain injury (TBI) according to neurologic outcomes.

**Variables**	**Good outcome (*n =* 41) GOSE 7 or 8**	**Poor outcome (*n =* 29)** **GOSE ≤6**	***p*-value**
**Clinical characteristics**
Male	35 (85.4%)	21 (72.4%)	0.182
Age, years	60.5 ± 19.1	62.7 ± 18.7	0.635
Hypertension	14 (34.1%)	13 (44.8%)	0.366
Diabetes mellitus	8 (19.5%)	7 (24.1%)	0.642
Hyperlipidemia	4 (9.8%)	3 (10.3%)	0.936
Smoking	13 (31.7%)	8 (27.6%)	0.711
**Neurologic and radiologic findings**
GCS score 9 or 10	8 (19.5%)	19 (65.5%)	<0.001
Significant abnormal CT findings	4 (9.8%)	15 (51.7%)	<0.001
Neuroworsening	6 (14.6%)	13 (44.8%)	0.005
**Laboratory results**
Hemoglobin (g/dL)	11.4 ± 1.2	11.1 ± 1.0	0.376
SaO_2_ (%)	94.4 ± 2.1	94.5 ± 1.6	0.776
Copeptin (pg/mL), median (IQR)	285.9 (254.6–313.2)	327.2 (290.2–349.9)	<0.001
C-reactive protein (mg/L)^*^, median (IQR)	14.0 (11.0–16.4)	17.3 (11.8–21.3)	0.019
**Treatment**
Craniotomy and hematoma removal	4 (9.8%)	11 (37.9%)	0.005

*Data show the numbers of subjects expressing discrete and categorical variables and mean ± SD.*

* *The normal value of C-reactive protein (CRP) was 0–5 mg/L.*

*Glasgow Outcome Scale Extended score 7 indicates minor physical or mental deficits or mental deficits that affects daily life and GOSE score 8 did full recovery or minor symptoms that do not affect daily life.*

*GCS, Glasgow Coma Scale; GOSE, Glasgow Outcome Scale Extended; and IQR, interquartile range*.

**Table 2:** Multivariable logistic regression analysis for 6-month poor outcome in patients with moderate TBI.

**Variables**	**Odds ratio**	**95% Confidence interval**	***p*-value**
GCS score 9 or 10	4.507	1.266–16.047	0.020
Significant abnormal CT findings	4.770	1.133–20.076	0.033
Copeptin	1.020	1.005–1.036	0.011
C-reactive protein	1.043	0.928–1.171	0.479
Neuroworsening	1.487	0.248–8.920	0.664
Craniotomy and hematoma removal	0.532	0.039–7.237	0.636

*CT, computed tomography; GCS, Glasgow Coma Scale*.

### Association Between Consecutive Plasma Copeptin and Neurologic Outcome

**Figure 2:**
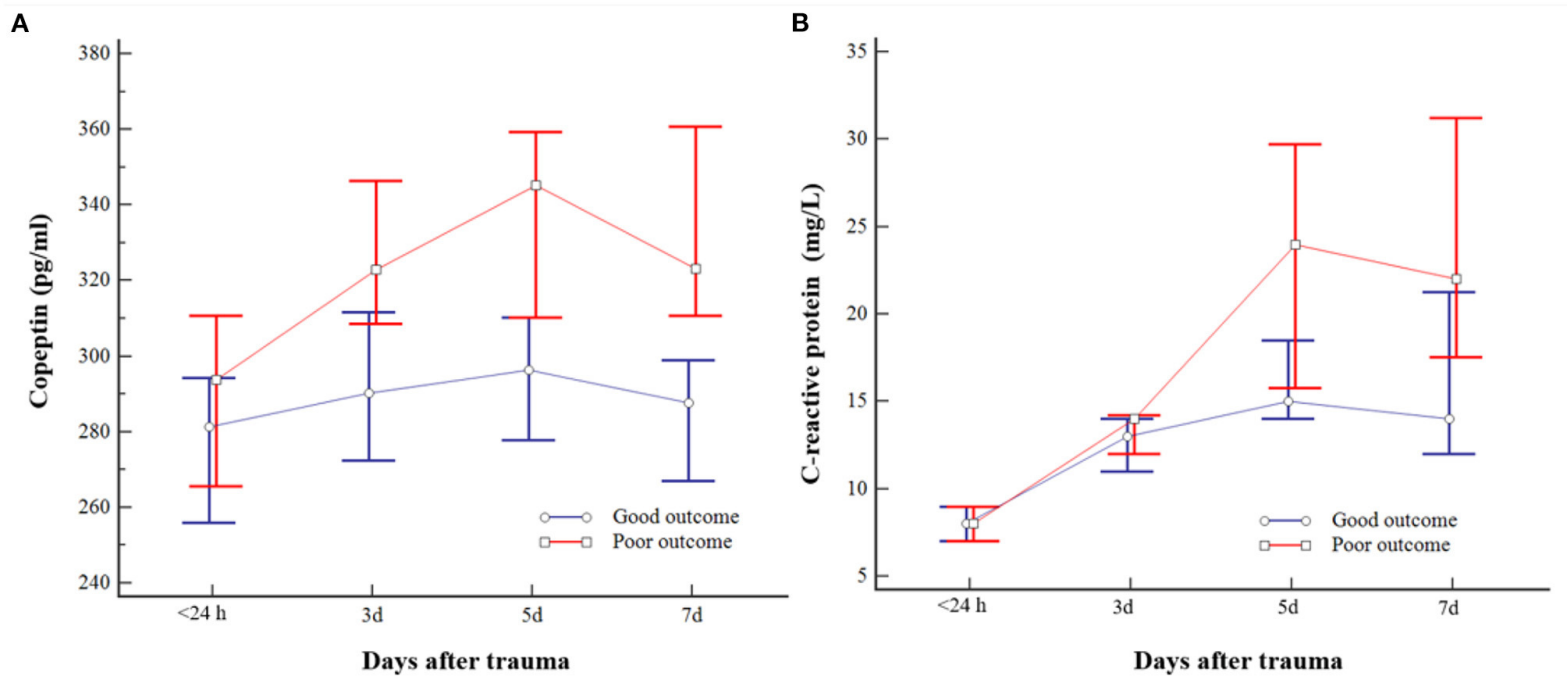
Changes in consecutive plasma copeptin **(A)** and C-reactive protein (CRP) **(B)** levels of patients with moderate traumatic brain injury (TBI). The bar indicates the median and 95% *CI*. Poor outcome following moderate TBI was closely related to copeptin measured on days 3, 5, and 7 and CRP measured on days 5 and 7, respectively.

The plasma copeptin level increased after injury and peaked on day 5 and decreased thereafter. Consecutive plasma copeptin levels were different significantly depending on the neurologic outcomes (*p* < 0.001) (Figure 2A). Difference in consecutive measured CRP was different significantly according to outcomes of patients over time (*p* = 0.002) (Figure 2B). Relationships between the measured values for each date and the outcomes were further analyzed. Except for difference in copeptin levels measured within 24 h, there was a significant difference in the copeptin level on day 3, 5, and 7, respectively. The specific results are as follows. Day 3 is [322.9 (283.4–360.7) pg/ml in the poor outcome and 290.3 (251.4–321.9) pg/ml in the good outcome; *p* = 0.010)], Day 5 [345.2 (307.4–380.9) pg/ml in the poor outcome and 296.3 (255.4–322.1) pg/ml in the good outcome; *p* < 0.001], and Day 7 [323.2 (308.8–380.6) pg/ml in the poor outcome and 287.7 (253.9–300.7) pg/ml in the good outcome; *p* < 0.001], respectively. The CRP levels measured on day 5 and 7 were significantly different according to outcomes.

The specific results are as follows. Day 5 [24.0 (14.7–32.2) mg/L in the poor outcome and 15.0 (13.0–22.0) mg/L in the good outcome (*p* = 0.014)] and day 7 [22.0 (13.7–33.5) mg/L in the poor outcome and 14.0 (10.0–22.0) mg/L in the good outcome (*p* = 0.015)], respectively. Given the above findings, copeptin was associated with outcomes from the 3rd day, whereas CRP may be associated with the outcomes from the 5th day in patients with moderate TBI.

### Prognostic Prediction Using Plasma Copeptin

**Figure 3:**
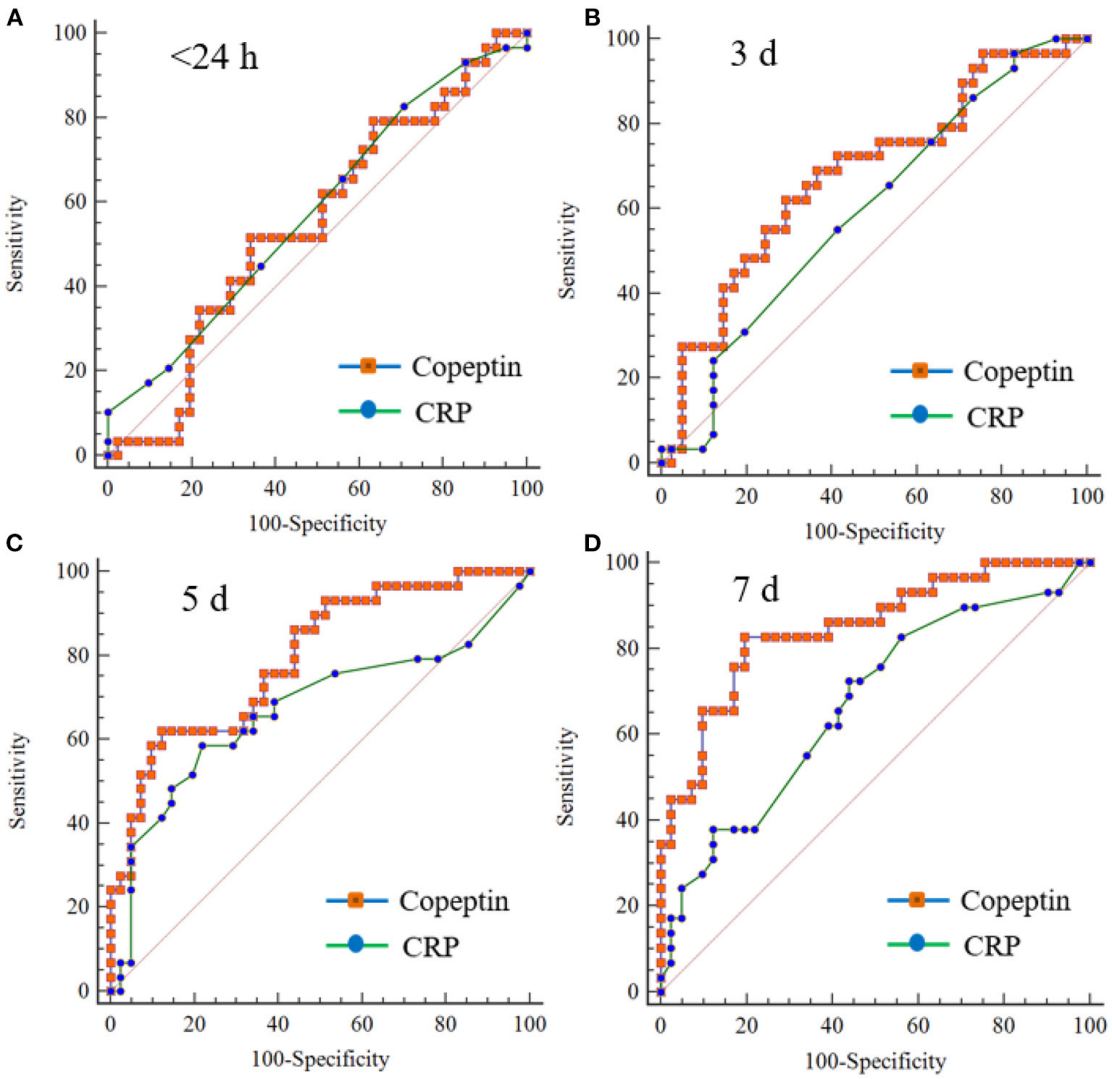
Comparison of receiver operating characteristic (ROC) curves between copeptin and CRP for predicting 6-month poor outcome in patients with moderate TBI. There is no significant difference in the comparison values between copeptin and CPR measured on within 24 h **(A)**, day 3 **(B)**, and day 5 **(C)**. The values measured on the 7th day **(D)** were statistically different (AUROC difference: 0.179, SE: 0.074, 95% *CI*: 0.032–0.325; *p* = 0.016). AUROC, area under the ROC curve; *CI*, confidence interval; and SE, standard error.

Predicting accuracy of poor outcome following moderate TBI according to the copeptin level measured on different days was assessed and compared using AUROC: day 3, AUROC = 0.681, 95% *CI*: 0.559–0.788; day 5, AUROC = 0.796, 95% *CI*: 0.682–0.883; and day 7, AUROC = 0.849, 95% *CI*: 0.743–0.923 (Supplementary Table S2). In particular, an increase in plasma copeptin > 300.8 pg/ml on day 7 exhibited a sensitivity of 82.76% (95% *CI*: 64.2–94.2%) and a specificity of 80.49% (95% *CI*: 65.1–91.2%) in predicting the poor outcome (Supplementary Figure S1). The level of CRP on day 5 and 7 was statistically significant in predicting the poor outcome: day 5, AUROC = 0.671, 95% *CI*: 0.548–0.779 (*p* = 0.016); and day 7, AUROC = 0.670, 95% *CI*: 0.547–0.778 (*p* = 0.011) (Supplementary Table S2). We further compared the prediction accuracy between plasma copeptin and CRP. Copeptin levels measured on day 7 showed better diagnostic performance than CRP with a AUROC difference of 0.179 (95% *CI*: 0.032–0.325, *p* = 0.016) (Figure 3 and Supplementary Table S3).

## Discussion

Prognostic factors related to neurologic outcomes after TBI has been investigated in gender, age, GCS score, pupillary reaction, abnormal findings on CT, and laboratory parameters, such as coagulopathy and anemia (18). In clinical practice, there is a tendency to predict outcome mainly based on GCS score and CT findings among various biomarkers. However, it is sometimes difficult to accurately predict outcomes merely relying on clinical factors and abnormalities on CT in actual clinical practice (19, 20). Moreover, judgements are sometimes interpreted differently depending on the examiner. Therefore, a robust biomarker is required and many studies have been performed to find biomarkers closely related to prognosis after TBI.

Plasma copeptin level was negatively correlated with GCS score, reflecting the degree of initial brain injury (7, 21). After brain injury, it increased, peaked in 24 h, showed plateau for 2 days and decreased gradually (21). Dong et al. (21) reported that the high plasma copeptin level was an independent risk factor for 1-month mortality with similar AUROC with GCS score in patients with severe TBI (Table 3). Additionally, copeptin was effective for the prediction of progressive hemorrhage injury and coagulopathy after severe TBI (22). However, Cavus et al. (23) did not find a significant difference in plasma levels measured at the time of admission and at 6 h according to survival status at 1 month after trauma. Rather, the difference between the copeptin at 6 h after trauma and that at admission was closely associated with the outcome. We thought that these slightly different results are thought to be due to heterogeneity derived from the TBI severity in the study. For mild TBI, copeptin remains controversial as a predictor of the poor outcome (19). Higher copeptin levels were observed in mild TBI compared with controls, but the association with abnormalities on CT and adverse events within a month was not clear. In the case of moderate TBI, it was analyzed together with patients with severe TBI, and there was no study on the role of copeptin in predicting outcomes in patients with moderate TBI. We first investigated the prognostic accuracy following moderate TBI. Our results revealed that increased plasma copeptin level was well correlated with poor neurologic outcomes in patients with moderate TBI.

**Table 3:** Summary of previous studies investigating the association between plasma copeptin and outcomes in patients with TBI.

**Study, year (Nation)**	**Indication**	**Sample size**	**Age**	**Detection method**	**Collection time**	**Relevance to copeptin**
Kleindienst et al. (GER) (7)	All TBI^§^	71	18–87^*^	ECLIA	Adm,3,7d	TBI severity, day 3 copeptin with low GOS
Dong et al. (CHN) (21)	Severe TBI	94	42.9 ± 18.6	ELISA	Adm,1,2,3,5,7 d	1-month mortality
Yu et al. (CHN) (24)	Severe TBI	106	45.4 ± 18.4	ELISA	Adm	12-month unfavorable outcome and mortality
Lin et al. (CHN) (25)	Severe TBI	126	8.0 ± 2.7	ECLIA	Adm	6-month unfavorable outcome and mortality
Cavus et al. (TUR) (23)	All TBI^¶^	53	1–92^*^	ELISA	Adm, 6 h	1-month mortality and mRS improvement^***^
Zhang et al. (CHN) (26)	Severe TBI	102	40.5 ± 15.3	ELISA	Adm	6-month unfavorable outcome and mortality
Yang et al. (CHN) (22)	Severe TBI	100	40.1 ± 15.1	ELISA	Adm	Coagulopathy and progressive hemorrhage
Castello et al. (ITA) (19)	Mild TBI	105	77 (59–83)^**^	IF	Adm	No association with 1-month mortality and readmission
Present et al. (KOR)	Moderate TBI	70	61.5 ± 18.9	ELISA	<24h, 3,5,7 d	6-month unfavorable outcome

*Age was presented as mean ± SD.*

* *indicates the range of the age in the study.*

** *indicates the median and interquartile of the age.*

*** *investigated the association between difference in Δ-Copeptin which were measured checked at admission and 6 h later, and outcomes.*

§ *included the severe TBI (n = 15), moderate TBI (n = 32), and mild TBI (24), respectively.*

¶ *included the severe TBI (n = 21), moderate TBI (n = 13), and mild TBI (19), respectively.*

*Adm, admission; ECLIA; electrochemiluminescence immunoassay; and ELISA, enzyme-linked immunosorbent assay*.

In this study, we compared the diagnostic accuracy for outcome prediction between copeptin and CRP in patients with moderate TBI. CRP is one of the acute phase reactants, but not brain-specific protein and reflects systemic inflammation (27–29). Xu et al. (1) reported that CRP increased up to 5 days after TBI and then decreased, and it became similar to the control group after 2 weeks. In particular, the CRP value measured at 2 weeks was highly correlated with the outcomes based on GOSE points (GOSE <5 vs. GOSE ≥ 5; AUROC = 0.892, 95% *CI*: 0.839–0.944). Even in patients with mild TBI, increased baseline CRP was associated with persistent post-concussion syndrome and persistent cognitive impairment (11). However, there are few reports on the association between CRP and moderate TBI. In our study, CRP on day 5 and 7 was significantly associated with poor neurologic outcomes. CRP over 18 measured on the 7th day showed high specificity of 87.80% (95% *CI*: 73.8–95.9%) in predicting poor outcomes. Although the prognostic accuracy of CRP is lower than copeptin, it can be a good alternative in situations where copeptin cannot be measured.

For TBI survivors, developing a tool that can be easily used in clinical practice and accurately predict long-term outcomes is necessary. Walker et al. (30) developed a clinical tool to provide information about the long-term functional outcomes in patients with moderate to severe TBI using national database. In their study, post-traumatic amnesia and GOS levels were strongly associated with prognosis. However, it is difficult to apply the prediction system made with simple clinical factors, such as productivity, number of prior TBI, occupation, problem alcohol use, and drug use to other hospitals with different medical environment. Moreover, their modeling method was not proven to be superior to the conventionally used regression analysis (31). We think that robust laboratory tests should be included in addition to important clinical factors to create a valid model that can be applied to other hospitals. Among several measurement days, copeptin measured on 7 days exhibited better predicting accuracy of 6-month poor outcome than initial significant abnormal findings on CT (AUROC difference = 0.139, 95% *CI*: 0.017–0.260, *p* = 0.024). In addition, it appeared to have better predicting accuracy compared with lower GCS score of 9 or 10 in patients with moderate TBI (AUROC = 0.119, 95% *CI*: −0.011 to 0.249, *p* = 0.07) (Supplementary Figure S2). Taking into account our findings, additional studies are required to create an easy-to-use model that can predict long-term outcomes based on clinical factors and laboratory tests for wider use in clinical practice.

The prognostic accuracy of copeptin has been widely investigated in various diseases, such as stroke, septic shock, and coronary artery disease, indicating that copeptin is not a specific biomarker for brain injury. Nevertheless, copeptin has advantages for clinical used of intracranial events assessment. Urwyler et al. (32) reported that copeptin directly reflects events in the brain and released into systemic circulation *via* bypassing the BBB. In addition, copeptin can be detected easily using automated assay within 30 min (20, 33). Two representative biomarkers, S100B and neuron-specific enolase (NSE), have been widely studied to assess the pathologic significance after TBI. It is not completely clear about the pathway where these biomarkers are found in serum. It is presumed that the biomarkers are secreted into extracellular space first, transported to the cerebrospinal fluid, then transferred to blood through passive diffusion (34). In addition, it is difficult to use them in the clinical practice based on the results obtained from different laboratories due to a discrepancy between serum S100B and albumin coefficient (34, 35). Accordingly, copeptin may be easily used in predicting the prognosis after TBI in the clinical practice due to easy to measure and to get consistent results than other biomarkers.

A strength of our study is that, for the first time, we investigated the association between copeptin and outcome in only patients with moderate TBI. Moreover, we compared the prediction accuracy with CRP which is widely used in the clinical practice and demonstrated better prediction accuracy than CRP. Accordingly, it became the rationale for using copeptin in patients with moderate TBI. However, our results warrant caution in interpretation for the following reasons. First, the number of the enrolled patients was 70 and such a relatively small sample size can be a concern to the interpretation. Second, pneumonia or septic conditions can increase copeptin level (36). We excluded patients with severe inflammation due to exacerbation of existing medical conditions within 72 h after admission, so it is difficult to generalize our results to patients whose underlying disease worsens. Third, we did not compare the prediction accuracy between copeptin and S100B or NSE. Zhang et al. (26) reported that plasma copeptin was the only marker that significantly improved prognostic value of GCS score, but not S100B or NSE in patients with severe TBI. Nevertheless, diagnostic superiority among biomarkers was not demonstrated in patients with moderate TBI. Fourth, we did not specifically classified abnormalities after TBI according to the existing scoring system, particularly Rotterdam CT score (37). Although the association of 6-month outcome in patients having moderate TBI with Rotterdam CT score was analyzed, no significant association was observed. As previously described, the classification of Rotterdam score into five categories for 70 patients with moderate TBI might have led to insufficient samples to draw a clear conclusion. In addition, due to differences in the severity of enrolled patients, it might be difficult to predict the prognosis of patients with moderate TBI with an initial Rotterdam CT score. Huang et al. (37) reported that Rotterdam CT score provided the great prognostic discrimination in patients who underwent decompressive craniectomy. Accordingly, it is necessary to construct an optimal prognostic model by comparing various CT classifications for many patients with moderate TBI in the future.

## Conclusion

Our results suggest that plasma copeptin can be helpful to predict 6-month outcomes in patients with moderate TBI.

## Data Availability Statement

The raw data supporting the conclusions of this article will be made available by the authors, without undue reservation.

## Ethics Statement

The studies involving human participants were reviewed and approved by Chuncheon Sacred Heart Hospital. The patients/participants provided their written informed consent to participate in this study.

## Author Contributions

JJ and SKa: contributed to conception and design of the study. SKi, TK, SH, SL, DY, and BK: performed experiment and organized the database. EH, CP, and J-TK: interpreted the results. JA, JR, JP, and HK: wrote the initial manuscript. All authors contributed to manuscript revision, read, and approved the submitted version.

## Funding

This research was supported by the National Research Foundation of Korea funded by the Ministry of Education (2020R1l1A3070726) and a grant of the Korea Health Technology R&D Project through the Korea Health Industry Development Institute (KHIDI) funded by the Ministry of Health & Welfare, Republic of Korea (grant number: HR21C0198). This work was supported by a research grant from Jeju National University Hospital in 2017.

## Conflict of Interest

The authors declare that the research was conducted in the absence of any commercial or financial relationships that could be construed as a potential conflict of interest.

## Publisher's Note

All claims expressed in this article are solely those of the authors and do not necessarily represent those of their affiliated organizations, or those of the publisher, the editors and the reviewers. Any product that may be evaluated in this article, or claim that may be made by its manufacturer, is not guaranteed or endorsed by the publisher.

## References

[1] Xu LB Yue JK Korley F Puccio AM Yuh EL Sun X. High-sensitivity c-reactive protein is a prognostic biomarker of six-month disability after traumatic brain injury: Results from the track-tbi study. J Neurotrauma. (2021) 38:918–27. 10.1089/neu.2020.7177 33161875 PMC7987360

[2] Kumar A Loane DJ. Neuroinflammation after traumatic brain injury: Opportunities for therapeutic intervention. Brain Behav Immun. (2012) 26:1191–201. 10.1016/j.bbi.2012.06.008 22728326

[3] Einarsen CE van der Naalt J Jacobs B Follestad T Moen KG Vik A. Moderate traumatic brain injury: clinical characteristics and a prognostic model of 12-month outcome. World Neurosurg. (2018) 114:e1199–210. 10.1016/j.wneu.2018.03.176 29614364

[4] Compagnone C d'Avella D Servadei F Angileri FF Brambilla G Conti C. Patients with moderate head injury: A prospective multicenter study of 315 patients. Neurosurgery. (2009) 64:690–6. 10.1227/01.NEU.0000340796.18738.F7 19197220

[5] Gan ZS Stein SC Swanson R Guan S Garcia L Mehta D. Blood biomarkers for traumatic brain injury: a quantitative assessment of diagnostic and prognostic accuracy. Front Neurol. (2019) 10:446. 10.3389/fneur.2019.00446 31105646 PMC6498532

[6] Klose M Juul A Struck J Morgenthaler NG Kosteljanetz M Feldt-Rasmussen U. Acute and long-term pituitary insufficiency in traumatic brain injury: A prospective single-centre study. Clin Endocrinol. (2007) 67:598–606. 10.1111/j.1365-2265.2007.02931.x 17880406

[7] Kleindienst A Brabant G Morgenthaler NG Dixit KC Parsch H Buchfelder M. Following brain trauma, copeptin, a stable peptide derived from the avp precusor, does not reflect osmoregulation but correlates with injury severity. Acta Neurochir Suppl. (2010) 106:221–4. 10.1007/978-3-211-98811-4_41 19812953

[8] Zhang J Wang H Li Y Zhang H Liu X Zhu L. The diagnosis and prognostic value of plasma copeptin in traumatic brain injury: a systematic review and meta-analysis. Neurol Sci. (2021) 42:539–51. 10.1007/s10072-020-05019-8 33389249

[9] ParkJJKimBJYounDHChoiHJJeonJP. A preliminary study of the association between sox17 gene variants and intracranial aneurysms using exome sequencing. J Korean Neurosurg Soc. (2020) 63:559–65. 10.3340/jkns.2019.022532380586

[10] Morris GF Juul N Marshall SB Benedict B Marshall LF. Neurological deterioration as a potential alternative endpoint in human clinical trials of experimental pharmacological agents for treatment of severe traumatic brain injuries. Executive committee of the international selfotel trial. Neurosurgery. (1998) 43:1369–72. 10.1227/00006123-199812000-00063 9848851

[11] Su SH Xu W Li M Zhang L Wu YF Yu F. Elevated c-reactive protein levels may be a predictor of persistent unfavourable symptoms in patients with mild traumatic brain injury: A preliminary study. Brain Behav Immun. (2014) 38:111–7. 10.1016/j.bbi.2014.01.009 24456846

[12] Rhim JK Youn DH Kim BJ Kim Y Kim S Kim HC. The role of consecutive plasma copeptin levels in the screening of delayed cerebral ischemia in poor-grade subarachnoid hemorrhage. Life. (2021) 11:274. 10.3390/life11040274 33806226 PMC8066417

[13] Xiao L Ma M Gu M Han Y Wang H Zi W. Renal impairment on clinical outcomes following endovascular recanalization. Neurology. (2020) 94:e464–73. 10.1212/WNL.0000000000008748 31857435

[14] Cebral JR Mut F Weir J Putman CM. Association of hemodynamic characteristics and cerebral aneurysm rupture. AJNR Am J Neuroradiol. (2011) 32:264–70 10.3174/ajnr.A2274 PMC3070915 21051508

[15] Jeon JS Kim JE Chung YS Oh S Ahn JH Cho WS. A risk factor analysis of prospective symptomatic haemorrhage in adult patients with cerebral cavernous malformation. J Neurol Neurosurg Psychiatry. (2014) 85:1366–70. 10.1136/jnnp-2013-306844 24681702

[16] Jeon JS Sheen SH Hwang G Kang SH Heo DH Cho YJ. Intravenous magnesium infusion for the prevention of symptomatic cerebral vasospasm after aneurysmal subarachnoid hemorrhage. J Korean Neurosurg Soc. (2012) 52:75–9. 10.3340/jkns.2012.52.2.75 23091662 PMC3467379

[17] Jeon JP Kim C Oh BD Kim SJ Kim YS. Prediction of persistent hemodynamic depression after carotid angioplasty and stenting using artificial neural network model. Clin Neurol Neurosurg. (2018) 164:127–31. 10.1016/j.clineuro.2017.12.005 29223792

[18] Amagasa S Tsuji S Matsui H Uematsu S Moriya T Kinoshita K. Prognostic factors of acute neurological outcomes in infants with traumatic brain injury. Childs Nerv Syst. (2018) 34:673–80. 10.1007/s00381-017-3695-4 29249074

[19] Castello LM Salmi L Zanotti I Gardino CA Baldrighi M Settanni F. The increase in copeptin levels in mild head trauma does not predict the severity and the outcome of brain damage. Biomark Med. (2018) 12:555–63. 10.2217/bmm-2018-0041 29620422

[20] Choi KS Cho Y Jang BH Kim W Ahn C Lim TH. Prognostic role of copeptin after traumatic brain injury: a systematic review and meta-analysis of observational studies. Am J Emerg Med. (2017) 35:1444–50. 10.1016/j.ajem.2017.04.038 28545954

[21] Dong XQ Huang M Yang SB Yu WH Zhang ZY. Copeptin is associated with mortality in patients with traumatic brain injury. J Trauma. (2011) 71:1194–8. 10.1097/TA.0b013e31821283f2 21502880

[22] Yang DB Yu WH Dong XQ Du Q Shen YF Zhang ZY. Plasma copeptin level predicts acute traumatic coagulopathy and progressive hemorrhagic injury after traumatic brain injury. Peptides. (2014) 58:26–9. 10.1016/j.peptides.2014.05.015 24905622

[23] Cavus UY Yildirim S Gurer B Dibek K Yilmaz D Ozturk G. The prognostic value of plasma delta-copeptin levels in patients with isolated traumatic brain injury. Eur J Trauma Emerg Surg. (2014) 40:373–8. 10.1007/s00068-013-0357-4 26816074

[24] Yu GF Huang Q Dai WM Jie YQ Fan XF Wu A. Prognostic value of copeptin: one-year outcome in patients with traumatic brain injury. Peptides. (2012) 33:164–9. 10.1016/j.peptides.2011.11.017 22138140

[25] Lin C Wang N Shen ZP Zhao ZY. Plasma copeptin concentration and outcome after pediatric traumatic brain injury. Peptides. (2013) 42:43–7. 10.1016/j.peptides.2013.01.015 23402790

[26] Zhang ZY Zhang LX Dong XQ Yu WH Du Q Yang DB. Comparison of the performances of copeptin and multiple biomarkers in long-term prognosis of severe traumatic brain injury. Peptides. (2014) 60:13–7. 10.1016/j.peptides.2014.07.016 25076464

[27] Adrian H Mårten K Salla N Lasse V. Biomarkers of traumatic brain injury: Temporal changes in body fluids. Eneuro. (2016) 3:ENEURO.0294-16.2016. 10.1523/ENEURO.0294-16.2016 28032118 PMC5175263

[28] Kim DY Cho KC. Extremely low serum alanine transaminase level is associated with all-cause mortality in the elderly after intracranial hemorrhage. J Korean Neurosurg Soc. (2021) 64:460–8. 10.3340/jkns.2020.0212 33626855 PMC8128522

[29] Lee Y Lim J Choi SW Han S Park B Youm JY. Changes of biomarkers before and after antibiotic treatment in spinal infection. Korean J Neurotrauma. (2019) 15:143-9. 10.13004/kjnt.2019.15.e19 PMC6826089 31720268

[30] Walker WC Stromberg KA Marwitz JH Sima AP Agyemang AA Graham KM. Predicting long-term global outcome after traumatic brain injury: development of a practical prognostic tool using the traumatic brain injury model systems national database. J Neurotrauma. (2018) 35:1587–95. 10.1089/neu.2017.5359 29566600 PMC6016099

[31] Hukkelhoven CW Rampen AJ Maas AI Farace E Habbema JD Marmarou A. Some prognostic models for traumatic brain injury were not valid. J Clin Epidemiol. (2006) 59:132–43. 10.1016/j.jclinepi.2005.06.009 16426948

[32] Urwyler SA Schuetz P Fluri F Morgenthaler NG Zweifel C Bergmann A. Prognostic value of copeptin: One-year outcome in patients with acute stroke. Stroke. (2010) 41:1564–7. 10.1161/STROKEAHA.110.584649 20508186

[33] Morgenthaler NG Struck J Alonso C Bergmann A. Assay for the measurement of copeptin, a stable peptide derived from the precursor of vasopressin. Clin Chem. (2006) 52:112–9. 10.1373/clinchem.2005.060038 16269513

[34] Dadas A Washington J Diaz-Arrastia R Janigro D. Biomarkers in traumatic brain injury (tbi): a review. Neuropsychiatr Dis Treat. (2018) 14:2989–3000. 10.2147/NDT.S125620 30510421 PMC6231511

[35] Kleindienst A Schmidt C Parsch H Emtmann I Xu Y Buchfelder M. The passage of s100b from brain to blood is not specifically related to the blood-brain barrier integrity. Cardiovasc Psychiatry Neurol. (2010) 2010:801295. 10.1155/2010/801295 20671945 PMC2910463

[36] Katan M Muller B Christ-Crain M. Copeptin: A new and promising diagnostic and prognostic marker. Crit Care. (2008) 12:117. 10.1186/cc6799 18355399 PMC2447549

[37] Huang YH Deng YH Lee TC Chen WF. Rotterdam computed tomography score as a prognosticator in head-injured patients undergoing decompressive craniectomy. Neurosurgery. (2012) 71:80–5. 10.1227/NEU.0b013e3182517aa1 22382208

