# Supplementary material for: Association Between Copeptin and Six-Month Neurologic Outcomes in Patients With Moderate Traumatic Brain Injury
Source: Front Neurol. 2022 Apr 25;12:749110. doi: 10.3389/fneur.2021.749110 (PMC9081440; doi:10.3389/fneur.2021.749110)
Supplement: Supplementary file 1 [file Data_Sheet_1.docx]

**Supplemental data**

**Supplemental Table S1.** Protocol for interpretation and inter-assessor agreements of CT findings.

1^st^ Reviewer, a neurosurgeon with more than 20 years of experience.

2^nd^ Reviewer, a neurosurgeon with more than 15 years of experience.

3^rd^ Reviewer, a radiologist with more than 25 years of experience.

CT findings were independently reported, and followed by a comparison of their interpretation of records. All disagreements were resolved by third reviewer. The Cohen’s kappa was 0.857, indicating almost perfect agreement.

| n = 70 | | 1^st^ Reviewer | |
| --- | --- | --- | --- |
|  |  | A | B |
| 2^nd^ Reviewer | A | 32 | 2 |
|  | B | 3 | 33 |

**Supplemental Table S2.** Predictive performance of consecutive measurements of plasma copeptin on 6-month poor outcome following moderate traumatic brain injury. Predictive performance of C-reactive protein (CRP) is also analyzed. AUROC, area under the ROC curve; SE, standard error; CI, confidence interval.

| Variable | Poor outcome prediction | AUROC | SE | 95% CI |
| --- | --- | --- | --- | --- |
| Copeptin | <24 hr | 0.549 | 0.07 | 0.426-0.668 |
|  | 3 d | 0.681 | 0.066 | 0.559-0.788 |
|  | 5 d | 0.796 | 0.054 | 0.682-0.883 |
|  | 7 d | 0.849 | 0.047 | 0.743-0.923 |
| CRP | <24 hr | 0.579 | 0.069 | 0.455-0.696 |
|  | 3 d | 0.598 | 0.068 | 0.473-0.713 |
|  | 5 d | 0.671 | 0.071 | 0.548-0.779 |
|  | 7 d | 0.670 | 0.066 | 0.547-0.778 |

**Supplemental Table S3.** Comparison of receiver operating characteristic (ROC) curves in predicting poor neurologic outcome at 6 months following moderate traumatic brain injury between copeptin and C-reactive protein. The difference between AUROC curve on day 7 was 0.179 (95% CI: 0.032-0.325; p=0.016). AUROC, area under the ROC curve; SE, standard error; CI, confidence interval.

| Measurement date | AUROC difference | SE | 95% CI | p-value |
| --- | --- | --- | --- | --- |
| <24 hr | 0.029 | 0.089 | -0.145-0.204 | 0.737 |
| 3 d | 0.083 | 0.095 | -0.103-0.270 | 0.378 |
| 5 d | 0.124 | 0.083 | -0.039-0.288 | 0.136 |
| 7 d | 0.179 | 0.074 | 0.032-0.325 | 0.016 |

**Supplemental Figure S1.** The area under the receiver operator characteristics curve is 0.849. The increased plasma copeptin more than 300.8 pg/ml on day 7 showed a sensitivity of 82.76% (95% CI: 64.2%-94.2%) and a specificity of 80.49% (95% CI: 65.1%-91.2%). CI= confidence interval. Orange-colored points correspond to criterion values. The blue-dotted lines indicate inclusion of 95% confidence bounds.


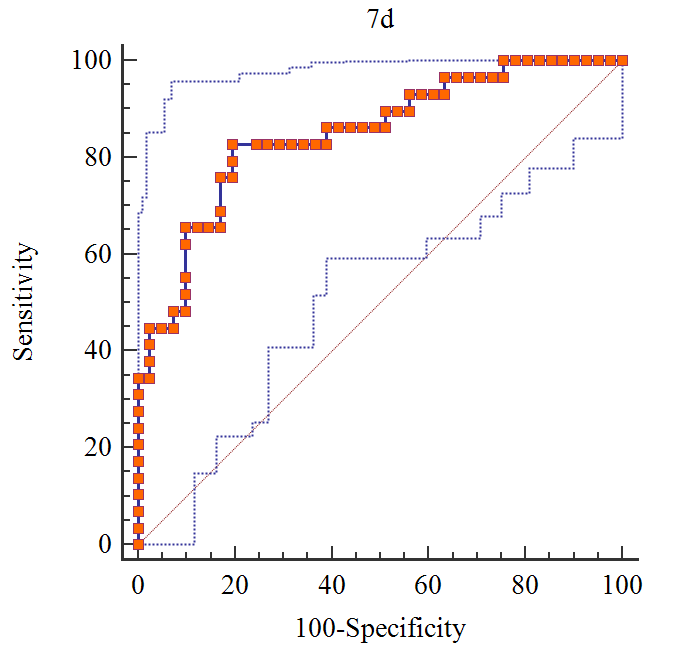


**Supplemental Figure S2.** Comparison of receiver operating characteristic (ROC) curves between copeptin at 7 days, initial GCS score 9 or 10, and significant abnormal CT findings in predicting 6-month poor outcome following moderate traumatic brain injury (TBI). There is no significant difference in the comparison values measured on within 24 hours (A), day 3 (B), and day 5 (C), and GCS score of 9 or 10, and significant abnormal findings on computed tomography (CT). AUROC, Area under the ROC curve; CI, confidence interval; SE, standard error.


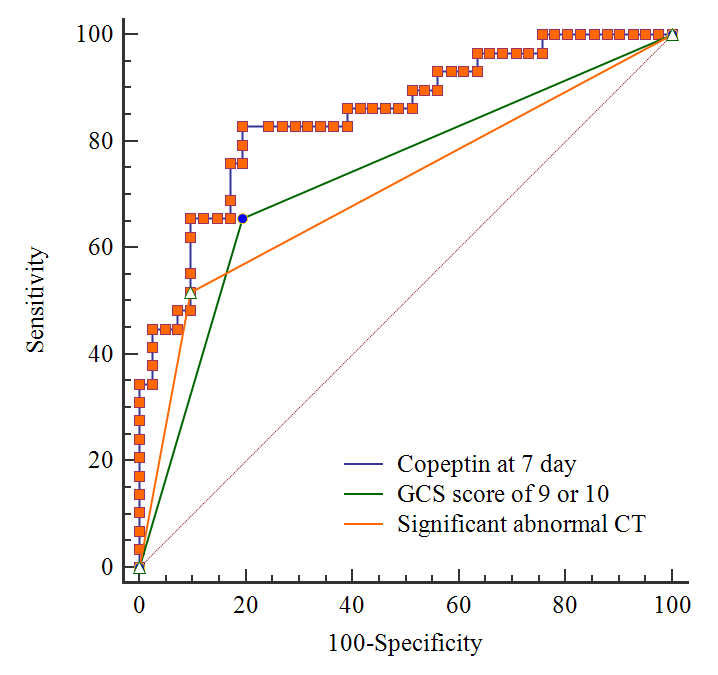


| Methods | AUROC | SE | 95% CI |
| --- | --- | --- | --- |
| Copeptin at 7 days | 0.849 | 0.047 | 0.743-0.923 |
| GCS score of 9 or 10 | 0.730 | 0.054 | 0.611-0.829 |
| Significant abnormal CT findings | 0.710 | 0.052 | 0.589-0.812 |
